# Development and validation of the quality care questionnaire –palliative care (QCQ-PC): patient-reported assessment of quality of palliative care

**DOI:** 10.1186/s12904-018-0296-2

**Published:** 2018-03-05

**Authors:** Young Ho Yun, Eun Kyo Kang, Jihye Lee, Jiyeon Choo, Hyewon Ryu, Hye-min Yun, Jung Hun Kang, Tae You Kim, Jin-Ah Sim, Yaeji Kim

**Affiliations:** 1 Department of Family Medicine, Seoul National University Hospital, 103 Daehak-ro, Jongno-GU, Seoul, 03080 Republic of Korea; 2 Department of Biomedical Informatics, Seoul National University College of Medicine, Seoul, South Korea; 3 Department of Biomedical Science, Seoul National University College of Medicine, Seoul, South Korea; 4 Department of Internal Medicine, Chungnam National University Hospital, Daejeon, South Korea; 5 Department of Internal Medicine, Postgraduate Medical School, Gyeongsang National University, Jinju, South Korea; 6 Department of Internal Medicine, Seoul National University Hospital, Seoul, South Korea

**Keywords:** Quality of care, Questionnaire, Palliative care, Validation

## Abstract

**Background:**

In this study, we aimed to develop and validate an instrument that could be used by patients with cancer to evaluate their quality of palliative care.

**Methods:**

Development of the questionnaire followed the four-phase process: item generation and reduction, construction, pilot testing, and field testing. Based on the literature, we constructed a list of items for the quality of palliative care from 104 quality care issues divided into 14 subscales. We constructed scales of 43 items that only the cancer patients were asked to answer. Using relevance and feasibility criteria and pilot testing, we developed a 44-item questionnaire. To assess the sensitivity and validity of the questionnaire, we recruited 220 patients over 18 years of age from three Korean hospitals.

**Results:**

Factor analysis of the data and fit statistics process resulted in the 4-factor, 32-item Quality Care Questionnaire-Palliative Care (QCQ-PC), which covers appropriate communication with health care professionals (ten items), discussing value of life and goals of care (nine items), support and counseling for needs of holistic care (seven items), and accessibility and sustainability of care (six items). All subscales and total scores showed a high internal consistency (Cronbach alpha range, 0.89 to 0.97). Multi-trait scaling analysis showed good convergent (0.568–0.995) and discriminant (0.472–0.869) validity. The correlation between the total and subscale scores of QCQ-PC and those of EORTC QLQ-C15-PAL, MQOL, SAT-SF, and DCS was obtained.

**Conclusion:**

This study demonstrates that the QCQ-PC can be adopted to assess the quality of care in patients with cancer.

**Electronic supplementary material:**

The online version of this article (10.1186/s12904-018-0296-2) contains supplementary material, which is available to authorized users.

## Background

Despite advances in cancer therapy for cancer, most patients with cancer still suffer due to the disease itself, as well as its treatment [1–3]. Patients with cancer have fatigue, pain, loss of appetite, depression, and social and spiritual distress [1]. Considering these various aspects, the definition of quality of life (QOL) in cancer patients is included personal insights of cancer patients’ symptoms, including physical, mental, social, and cognitive functions [4]. As attention to QOL in oncologic care throughout the cancer trajectory has increased, palliative care is being better integrated into oncologic care at earlier stages, than in the past [1–3].

The World Health Organization (WHO) defines palliative care as “an approach that improves the QOL of patients and their families facing the problems associated with life-threatening illness, through the prevention and relief of suffering by means of early identification and impeccable assessment and treatment of pain and other problems, physical, psychosocial, and spiritual”. Recently, it was demonstrated that early palliative care resulted in not only a significant improvement in QOL [5–7], but also longer survival among patients with cancer [5, 8].

Because palliative care is one of the factors that influences the patient’s QOL, it is also important that the patient is provided with palliative care of good quality defined by two crucial, humane dimensions: high quality of care, and full acceptance of the patient’s intentions so that care is provided in a humane and culturally appropriate manner [9].

To design palliative care intervention strategies tailored to the patient, it is crucial to identify patients at a high risk for poor quality care by using a valid assessment tool. However, most instruments measure quality of care in patients at end of life (EOL) [10–15]. There are few assessment tools focusing on quality of care in patients with cancer receiving palliative care [16, 17], and there are limitations in that most of the current palliative care assessment tools did not include spiritual and cultural domains, and there are few tools to assess the EOL care experience reported by the patient [18]. In addition, most of the palliative care assessment tools developed so far measure the QOL of the patient, not the quality of care [19–23].

Therefore, in the present study, we aimed to develop a new instrument to assess quality of palliative care and report on the validation of the developed tool, which is called Quality of Care Questionnaire-Palliative Care scale (QCQ-PC).

## Methods

### Study design

QCQ-PC includes four stages of development proposed by the European Organization for Research and Treatment of Cancer Quality of-Life Group [24]: 1) item generation and reduction; 2) scale construction; 3) pilot testing; and 4) field testing. The study was approved by the Institutional Review Board of the Seoul National University Hospital, Korea. Suitable patients signed informed consent forms. The requirements for the recruitment of patients were as follows: (1) 18 years old or older; (2) diagnosed with cancer (cancer was confirmed by oncologists); (3) able to read and comprehend Korean, be able to fill in the questionnaire; and (4) aware of cancer diagnosis.

### Phase I: Item generation and reduction

Phase I was intended to gather a list of relevant quality of palliative care questionnaires and previous palliative care clinical guidelines. We performed an extensive literature review using PUBMED and other databases searching the keywords ‘palliative care’, ‘end of life care’, and ‘quality of care’. We conclusively reviewed and adopted End of Life Quality Standards (2013) published by The National Institute for Health and Care Excellence (NICE) in the United Kingdom, Clinical Practice Guidelines for Quality Palliative Care (3rd Ed. 2013) released by National Consensus Project (NCP) in the United States and A National Framework and Preferred Practices for Palliative and Hospice Care Quality issued by National Quality Forum (NQF) in the United States.

We also considered the quality of care index of the prior study. We investigated and used the Assessment of Chronic Illness Care (ACIC) questionnaire and the Patient Assessment of Chronic Illness Care (PACIC) questionnaire translated by National Evidence-based Healthcare Collaborating Agency (NECA) in a previous study, which was a study of the performance evaluation methods of sub-categories in the management of chronic disease patients in a community by the primary health care center (2015). In addition, the FAMCARE Patient Scale developed by Kristjanson (1993) and verified with respect to validity and reliability was used. Based on the literature, we constructed a list of items for the QCQ-PC from 104 quality care issues divided into 14 scales.

We discussed these 104 items in semi-structured interviews with 31 health care professionals in November 2016 and December 2016. The health care professionals participating in the survey included physicians, nurses, and social workers who worked in university hospitals and were engaged in palliative care. The semi-structured interviews did not include patients because the items built by healthcare professionals were intended to be pilot-tested separately. The survey was conducted twice in total, and the respondents evaluated the validity and feasibility of the developed items by scoring them on a scale from 1 to 5. In the second investigation, the average score of all respondents for each item was compared with the score given by them, and the score changed when the respondent’s evaluation changed. The items that did not meet the following criteria in both the primary and secondary surveys were deleted (in the case of validity, if the respondent average score was three or more, or the respondent score was less than three points in less than 25% of the respondents; in the case of feasibility, the respondent average score is 2.5 or more, or less than three points in less than 30% of the total respondents). Thirteen items (12.5% of the developed items) that did not meet the criteria were deleted; therefore, a total of 91 items were included in the final index.

### Phase II: Scale construction

Among the 91 palliative care quality evaluation indices developed through the process of item development and reduction, we constructed scales of 43 items that only the patients were asked to answer. The 48 items that were excluded were the items that directly asked the medical staff to evaluate the quality of care. For the scoring format, we selected a four-point Likert scale for all of 43 items to evaluate in phase III (Strongly Agree, Agree, Disagree, Strongly Disagree – see Additional file 1: Appendix B).

### Phase III: Pilot testing

We conducted pilot tests to find possible administration problems, such as miss-phrasing, and to determine which items should be modified or removed. Before field testing, a total of 15 cancer patients responded to the QCQ-PC questionnaire and debriefed the questionnaire. The mean age of the 15 patients was 60.6 years, with 7 males and 8 females. Regarding educational level, most subjects had more than college or university education. All 15 patients were collected from Seoul National University Hospital. All 15 patients were collected from Seoul National University Hospital. The questionnaire designed for debriefing included which questions were misleading, whether the respondents were offended, or whether they were unable to respond. After the questionnaire, we received comments on the questionnaire. Taking into consideration the responses of these debriefing questionnaires, we clarified and simplified the items that were difficult to understand or answer. We also added one item to reflect the needs of the respondents.

### Phase IV: Field testing

To confirm reliability and validity, we performed a field test with the QCQ-PC questionnaire.

Because the most patients group receiving palliative care are patients diagnosed with cancer, we performed this field testing for the QCQ-PC questionnaire to cancer patients. To obtain a heterogeneous sample by cancer type, patients were recruited to include various primary sites of cancer; these patients were registered at four university hospitals in Korea. We included patients under curative treatment as well as patients under palliative treatment. This study used factor analysis with varimax rotation to analyze construct validity, and performed multi-trait scaling analysis to examine the extent to which QCQ-PC items could be combined into a more controlled multi-item set. For validation, we applied ten rules, for which at least five respondents were required per item [25, 26]. Since we had a total of 44 questions, 220 respondents were necessary for the validation process.

We investigated the correlation between each question and the scale that included that question, and estimated the convergent validity of the QCQ-PC items*.* In verifying discriminant validity, we assessed the magnitude of the correlation of an item with its own scale as compared to other scales. We considered the convergent validity of items by analyzing correlations that were ≥0.4, and corrected for overlap, as confirmation of validity [27]. We identified scaling errors as cases in which an item correlated significantly less with its own scale as compared to its correlation with other scales. To test the reliability of QCQ-PC, we estimated Cronbach alpha, a degree of internal consistency of responses. An alpha ≥0.70 was generally regarded as acceptably high for the collection of responses into a single score [28].

Furthermore, fit statistics (INFIT/OUTFIT) analysis was performed to evaluate the fitness of the item response, and it was interpreted as a good response when the fit was between 0.7 and 1.3 [29]. The slope was checked to confirm the discrimination of the items. In the case of a fit value less than 1.5, the discrimination of the items was considered unsuitable (see Additional file 1: Appendix A).

### Additional evaluation

We asked the respondents to fill in additional questionnaires on previous validated scales. The following questionnaires were included: The European Organization for Research and Treatment of Cancer Quality of Life Questionnaire Core-15 item palliative care (EORTC QLQ–C15-PAL) measuring patient-reported QoL [30], the McGill Quality of Life Questionnaire (MQOL) constructed by physical symptoms, psychological symptoms, existential well-being, and support [31], the Smart Management Strategy for Health Assessment Tool – Short Form (SAT-SF) for self-management strategies assessments [32], and the Decisional Conflict Scale (DCS) - assesses the level of ‘decisional conflict’ that patients experience while making health care decisions [33].

Data analyses were conducted using WINSTEP version 4.0 for item-fit analysis, and SPSS version 24.0 for other statistical analyses.

## Results

A total of 220 patients under curative or palliative therapy were enrolled in this study during the 2-month survey period. Most patients (96.4%) completed the questionnaire, but eight patients were excluded from the analysis because they did not answer more than three items. The remaining 212 patients included in the analysis responded to all questions.

The demographic characteristics of patients who participated in the study are shown in Table 1. There was no significant difference in gender ratio; more than half the patients were aged between 50 and 69 years old, and most patients were being treated.

**Table 1:** Demographic and clinical characteristics of the Participants (*N*=212)

Characteristics		N (%)
Age	< 29	6 (2.8)
	30–39	10 (4.7)
	40–49	42 (19.8)
	50–59	57 (26.9)
	60–69	59 (27.8)
	≥70	38 (17.9)
Sex	Male	100 (47.2)
	Female	112 (52.8)
Education	≤Elementary school	29 (13.8)
	Middle school	20 (9.5)
	High school	72 (34.1)
	≥College or University	90 (42.7)
Primary site^a^	Breast	54 (25.5)
	Lung	28 (13.2)
	Colon	29 (13.7)
	Blood	29 (13.7)
	Stomach	22 (10.4)
	Urinary system	17 (8.0)
	Pancreato-biliary system	16 (7.5)
	Others	17 (8.0)
Therapeutic status^b^	At diagnosis	4 (1.9)
	In treatment	162 (76.8)
	< 5 yrs. since treatment	31 (14.7)
	≥5 yrs. since treatment	14 (6.6)

^a^Primary site of cancer

^b^Therapeutic status of cancer

### Factor analysis

We conducted factor analysis on the total sample of respondents (*n* = 220). We initially had four significant factors with 44 items; twelve items were discarded through the fit statistics process. Upon reanalysis of the remaining 32 items, the items were classified according to four significant factors. Table 2 lists the item-to-factor correlations for the 32 items and four factors – factor 1: appropriate communication with health care professionals (ten items); factor 2: discussing value of life and goals of care (nine items); factor 3: support and counseling for needs of holistic care (seven items); and factor 4: accessibility and sustainability of care (six items). We attained similar of factor analysis results with both multiple and simple imputations.

**Table 2:** Factor analysis of 32 QCQ-PC Items

QCQ-PC items		Factor^b^			
No^a^	Questions	Factor 1	Factor 2	Factor 3	Factor 4
3	I am satisfied with the careful manner of medical staff	**0.767**	0.164	0.334	0.148
6	I am satisfied with the way of communication of medical staff	**0.755**	0.212	0.350	0.118
2	I was able to receive adequate care from medical staff	**0.742**	0.163	0.219	0.149
7	I have heard and understood an accurate description of the progress of my disease	**0.734**	0.304	0.105	0.186
11	The medical staff explained terms that I was curious about	**0.662**	0.245	0.326	0.219
4	I was able to receive the healthcare service I demanded	**0.634**	0.378	0.106	0.370
9	The medical staff support my decision on care plan	**0.601**	0.446	0.091	0.495
8	I have heard and understood an accurate description of my care plan	**0.600**	0.441	0.026	0.433
43	I was able to have a conversation with medical staff in a relaxed atmosphere	**0.583**	0.222	0.581	0.119
25	The medical staff paid attention to various symptoms I felt and adjusted them well	**0.580**	0.097	0.446	0.300
13	I was able to discourse with medical staff about the value of my life	0.305	**0.737**	0.375	0.044
14	I was able to recall what is important to achieve the values and goals of my life while discoursing with medical staff	0.250	**0.709**	0.432	0.032
16	I was able to express what my family and I expected from care	0.310	**0.704**	0.229	0.289
18	My care plans included the things I was able to try myself	0.141	**0.684**	0.115	0.336
19	I was able to receive adequate help from medical staff, while I was having difficulties in setting up specific goals related to care	0.196	**0.683**	0.324	0.358
17	My family and I received an education that is helpful to care	0.246	**0.670**	0.333	0.226
21	The medical staff suggested an adequate care plan in consideration of values of my life	0.303	**0.599**	0.289	0.434
22	I was able to modify my plan when my demand for treatment changed	0.279	**0.557**	0.274	0.395
12	The medical staff managed intermediate checkups to verify whether I could execute my goals	0.509	**0.544**	0.180	0.284
44	I was able to receive outpatient care and telephone counseling with plenty of time	0.325	0.293	**0.702**	0.123
30	The medical staff provide support to me and my family to solve spiritual concerns	0.043	0.360	**0.647**	0.225
29	The medical staff provided support to me and my family to overcome social crisis	0.224	0.451	**0.620**	0.262
33	The medical staff knew what I wanted	0.257	0.187	**0.619**	0.382
28	The medical staff communicated smoothly with me and my family	0.291	0.255	**0.592**	0.380
41	Outpatient care and telephone counseling were done at the appointed time without delay	0.483	0.138	**0.524**	0.078
27	My family and I received psychological support from medical staff	0.242	0.299	**0.508**	0.424
35	Services needed for my care are provided by experts in their respective fields	0.148	0.228	0.367	**0.706**
36	I was able to get care services at the locations I wanted	0.237	0.293	0.404	**0.669**
38	Medical care is immediately provided in a state of crisis	0.315	0.228	0.340	**0.552**
23	The medical staff periodically confirmed my goals and plans toward care	0.275	0.482	0.341	**0.540**
10	The decision on a healthcare plan was reflected by my family and my opinions	0.485	0.419	0.009	**0.517**
5	I understand the goal of care	0.429	0.450	0.151	**0.503**

Results from orthogonal (varimax) rotation analysis. Bold type indicates loading > 0.5

^a^The number of each item is its number in the QCQ-PC questionnaires

^b^Factor 1: Appropriate communication with health care professionals; Factor 2: Discussing value of life and goals of care; Factor 3: Support and counseling for needs of holistic care; Factor 4: Accessibility and sustainability of care

### Reliability

Table 3 shows the mean and reliability of the QCQ-PC subscale. In all four scales, the degree of reliability was high, with good internal consistency (Cronbach alpha range: 0.889–0.973).

**Table 3:** Descriptive statistics and subscale reliability results for QCQ-PC items

Subscales	Number of Items	Range of Scores	Mean(SD)	Reliability Cronbach’s α	Convergent Validity	Discriminant Validity	Scaling Success	Scaling Errors (%)
Total	32	0–100	2.813 (0.555)	0.973				
Factor 1: Appropriate communication with healthcare professionals	10	0–100	3.036 (0.576)	0.940	0.699–0.858	0.527–0.760	40/40	0
Factor 2: Discussing value of life and goals of care	9	0–100	2.688 (0.616)	0.935	0.691–0.852	0.514–0.740	36/36	0
Factor 3: Support and counseling for needs of holistic care	7	0–100	2.673 (0.597)	0.889	0.568–0.839	0.472–0.721	28/28	0
Factor 4: Accessibility and sustainability of care	6	0–100	2.866 (0.602)	0.896	0.655–0.885	0.512–0.869	24/24	0

*Abbreviations: QCQ-PC* Quality Care Questionnaire – Palliative Care, *SD* Standard deviation

### Validity: Multi-trait scaling analysis

When item-to-self scale correlations were tested, all item-convergent validity exceeded 0.4. Other scales were also compared through item-discriminant validity, and no scaling error was observed.

### Additional evaluation

#### Additional evaluationMQOL

We obtained the correlation between total and subscale scores of QCQ-PC and EORTC QLQ-C15-PAL and MQOL (see Table 4). Both total and subscale scores significantly correlated with the emotional functioning and global health scores of EORTC QLQ-C15-PAL (Pearson correlation [r] range 0.165–0.278). Existential well-being and support scores of MQOL significantly correlated with both the total and subscale scores of QCQ-PC (Pearson correlation [r] range 0.218–0.341, *p* < 0.001). However, total and subscale scores of QCQ-PC did not correlate with physical functioning.

**Table 4:** Pearson correlations of QCQ-PC with other validated questionnaires (EORTC QLQ-C15-PAL, MQOL, SAT-SF, DCS)

		QCQ-PC subscale				
		Total score	Factor 1	Factor 2	Factor 3	Factor 4
EORTC-PAL	Physical functioning	0.076	0.091	0.034	0.089	0.089
	Emotional functioning	0.229***	0.259***	0.165*	0.231***	0.236***
	Global health	0.240***	0.278***	0.185**	0.206**	0.242***
McGill QOL	Existential well-being	0.323***	0.341***	0.237***	0.292***	0.308***
	Support	0.305***	0.310***	0.218***	0.285***	0.299***
SAT-SF strategies	Total	0.363***	0.351***	0.285***	0.305***	0.372***
	Core strategies	0.304***	0.310***	0.239***	0.242***	0.321***
	Preparation strategies	0.375***	0.354***	0.301***	0.316***	0.373***
	Implementation strategies	0.344***	0.324***	0.261***	0.301***	0.354***
DCS	Total	−0.397***	−0.357***	−0.378***	−0.325***	−0.376***
	Uncertainty	−0.367***	−0.324***	− 0.347***	− 0.323***	− 0.348***
	Informed	− 0.282***	− 0.265***	− 0.251***	− 0.204**	− 0.275***
	Values clarity	− 0.267***	−0.200**	− 0.268***	−0.235***	− 0.241***
	Support	−0.384***	−0.366***	− 0.374***	−0.317***	− 0.353***
	Effective decision	−0.349***	−0.327***	− 0.329***	−0.270***	− 0.344***

*Abbreviation: EORTC QLQ-C15-PAL* European Organization for Research and Treatment of Cancer Quality of Life Questionnaire Core-15 item palliative care, *McGill QOL* McGill Quality of Life Questionnaire, *SAT* Smart Management Strategy for Health (SMASH) Assessment Tool, *DCS* Decisional Conflict Scale

*** *P* < 0.001, ** *p* < 0.01, * *p* < 0.05

#### Comparisons with SAT-SF (smart management strategy for health assessment tool – Short form) scores

SAT is a tool to identify self-management strategies of cancer patients. We used the short form of SAT to examine the association with the QCQ-PC score. As expected, there was a significant correlation between the SAT total score and the QCQ-PC score (Pearson correlation [r] range 0.285–0.372, *p* < 0.001). Compared to each subscale of SAT, significant correlation was observed (Pearson correlation [r] range 0.239–0.375, *p* < 0.001).

#### Comparisons with DCS

QCQ-PC scores showed correlation with the DCS total score and subscale score (Pearson correlation [r] range 0.325–0.397, 0.200–0.384). DCS is scored in the opposite direction; the higher the score, the higher the decision conflict.

## Discussion

The 32-item QCQ-PC is patient-reported and has excellent psychometric properties. The QCQ-PC consists of four factors that focus on communication with health care professionals, discussing value of life and goals of care, support and counseling for holistic care needs, and accessibility and continuity of care, all of which are central issues in palliative care. Our results are consistent with the findings of many previous studies and recommendations [1, 3, 6], but diverge in terms of quality of care at EOL [10–15]. The factors are unique and different from standard oncology care [6].

This study suggests that QCQ-PC has excellent psychometric properties, such as high construct validity and internal consistency. That QCQ-PC exhibited less correlation with EORTC QLQ-C15-PAL than with MQOL, and does not have significant correlation with physical functioning, suggesting that QCQ-PC more measures the emotional, social, and spiritual aspects of care in palliative settings rather than symptom control. Additionally, most correlations of QCQ-PC subscales with QOL subscales were below 0.4, suggesting that QCQ-PC may differentiate aspects of palliative care from those addressed by the QOL assessment tools. Therefore, QCQ-PC is recommended for use together with a QOL assessment tool, such as EORTC QLQ-C15-PAL, in order to more accurately to measure the quality of care.

In this study, QCQ-PC had significant negative correlation between the total and subscale scores and the DCS scores. Lack of communication with healthcare professionals and discussing value of life and goals of care may cause patient dissatisfaction with respect to care during cancer treatment, and lead patients to choose interventions that physicians believe are inappropriate. In addition, it is particularly crucial for healthcare providers to support and counsel on holistic care needs, to deliver continuous palliative care for patients with cancer, to maintain good interpersonal relationships with them, and to relieve their decisional conflict [1, 2, 6, 10]. We can improve their decision conflicts by recognizing high risk groups in quality care and providing palliative care.

Perhaps the most interesting finding of this study is that QCQ-PC appears to be sensitive to health management in palliative care settings. Using a conceptual model, self-health management with cancer can be influenced by quality care including communication with health care professionals, discussing value of life and goals of care, support and counseling for holistic care needs, and accessibility and continuity of care. Our findings showing that the QCQ-PC scales were associated with strategy scales for health management support this conceptual model.

### Study limitations

This study has several limitations. First, since our research was conducted only in Korea, cross-cultural validation studies are necessary for generaliz**at**ions to **other** countries. Second, we did not assess test-retest. As QOL and quality of care among cancer patients can frequently change, test-retest may not be feasible and may not limit the psychometric properties of this QCQ-PC. Finally, as this study relied exclusively on patients with cancer, our findings might not be generalizable to non-cancer patients who need palliative care. Furthermore, our study included not only incurable cancer patients, but also patients who had received treatment more than 5 years previously. However, to assess the quality of early palliative care as well, it may be preferable to include all cancer patients rather than only those with incurable cancer for constructing the questionnaire. We suggested that it is necessary to investigate further research on the patients of early palliative care and palliative care with this QCQ-PC questionnaire. Though, some modification of items among the QCQ-PC might be applied to patients with other advanced diseases.

## Conclusion

In conclusion, we believe that this QCQ-PC, a self-reported assessment tool with proper psychometric properties, can be effectively used to identify patients with cancer at high risk, and to evaluate the efficacy of trials, such as palliative care with the QOL assessment tool.

## Additional file

Additional file 1: **Appendix A**. Factor analysis (Item discrimination, slope, and factor loading) and fit statistics (INFIT/OUTFIT) analysis; Table describing the result of factor analysis and fit statistics of 44 items. **Appendix B**. Quality Care Questionnaire –Palliative Care (QCQ-PC); Palliative care quality questionnaire validated in this paper. (DOCX 50 kb)

## Abbreviations

CI: Confidence interval
DCS: Decisional conflict scale
EOL: End of life
EORTC QLQ-C15-PAL: European Organization for Research and Treatment of Cancer Quality of Life Questionnaire Core-15 item palliative care
MQOL: The McGill quality of life questionnaire
OR: Odds ratio
QCQ-PC: Quality care questionnaire-palliative care
QOL: Quality of life
SAT-SF: Smart management strategy for health assessment tool – short form
