# Supplementary material for: Development and validation of the quality care questionnaire –palliative care (QCQ-PC): patient-reported assessment of quality of palliative care
Source: BMC Palliat Care. 2018 Mar 5;17:40. doi: 10.1186/s12904-018-0296-2 (PMC5836356; doi:10.1186/s12904-018-0296-2)
Supplement: Supplementary file 1 — Appendix A. Factor analysis (Item discrimination, slope, and factor loading) and fit statistics (INFIT/OUTFIT) analysis; Table describing the result of factor analysis and fit statistics of 44 items. Appendix B. Quality Care Questionnaire –Palliative Care (QCQ-PC); Palliative care quality questionnaire validated in this paper. (DOCX 50 kb) [file 12904_2018_296_MOESM1_ESM.docx]

Additional file 1

Appendix A. Factor analysis (Item discrimination, slope, and factor loading) and fit statistics (INFIT/OUTFIT) analysis

| No | QCQ-PC Questions | Discrimination  (Pearson) | Discrimination  (Polyseria) | Slope | Factor loading | Infit | Outfit |
| --- | --- | --- | --- | --- | --- | --- | --- |
| 1 | I was able to receive treatment promptly. | **0.54^a^** | 0.61 | **1.57^c^** | 0.69 | **1.41^e^** | **2.57^f^** |
| 2 | I was able to receive adequate care from medical staff. | 0.66 | 0.74 | 2.16 | 0.77 | 0.85 | 0.99 |
| 3 | I am satisfied with the careful manner of medical staff. | 0.71 | 0.79 | 2.68 | 0.77 | 1.16 | 1.06 |
| 4 | I was able to receive the healthcare service I demanded. | 0.75 | 0.82 | 2.89 | 0.52 | 1.02 | 0.99 |
| 5 | I understand the goal of care. | 0.76 | 0.83 | 3.16 | 0.58 | 0.83 | 0.77 |
| 6 | I am satisfied with the way of communication of medical staff. | 0.73 | 0.80 | 2.70 | 0.74 | 1.09 | 1.01 |
| 7 | I have heard and understood an accurate description of the progress of my disease. | 0.68 | 0.76 | 2.35 | 0.63 | 1.04 | 1.05 |
| 8 | I have heard and understood an accurate description of my care plan. | 0.73 | 0.81 | 2.78 | 0.60 | 0.97 | 0.94 |
| 9 | The medical staff support my decision on care plan. | 0.79 | 0.86 | 3.54 | 0.67 | 0.94 | 0.89 |
| 10 | The decision on a healthcare plan was reflected by my family and my opinions. | 0.71 | 0.77 | 2.64 | 0.63 | 1.13 | 1.05 |
| 11 | The medical staff explained terms that I was curious about. | 0.72 | 0.81 | 2.85 | 0.66 | 0.84 | 0.81 |
| 12 | The medical staff have managed intermediate checkups to verify whether I could execute my goals. | 0.77 | 0.83 | 3.07 | 0.52 | 0.81 | 0.79 |
| 13 | I was able to discourse with the medical staff about the value of my life. | 0.74 | 0.79 | 2.45 | 0.61 | 0.95 | 0.97 |
| 14 | I was able to recall what is important to achieve the values and goals of my life while discoursing with the medical staff. | 0.74 | 0.79 | 2.43 | 0.55 | 0.95 | 0.96 |
| 15 | The medical staff confirmed how well my family and I understood the disease. | 0.73 | 0.78 | 2.60 | **0.49^d^** | 0.91 | 0.91 |
| 16 | I was able to express what my family and I expected from care. | 0.77 | 0.83 | 3.03 | 0.71 | 0.80 | 0.80 |
| 17 | My family and I received an education that is helpful to care. | 0.73 | 0.80 | 2.51 | 0.64 | 0.91 | 0.89 |
| 18 | My care plans included the things I was able to try myself. | 0.65 | 0.72 | 2.12 | 0.69 | 1.12 | 1.10 |
| 19 | I was able to receive adequate help from the medical staff when I had difficulties in setting up specific goals related to care. | 0.77 | 0.83 | 3.24 | 0.70 | 0.84 | 0.82 |
| 20 | The medical staff supported me to build up a care plan as I wanted. | 0.82 | 0.88 | 3.93 | 0.72 | **0.62^e^** | **0.62^f^** |
| 21 | The medical staff suggested an adequate care plan in consideration of values of my life. | 0.79 | 0.85 | 3.46 | 0.70 | 0.77 | 0.73 |
| 22 | I was able to modify my plan when my demand for treatment changed. | 0.72 | 0.78 | 2.62 | 0.56 | 0.93 | 0.91 |
| 23 | The medical staff periodically confirmed my goals and plans toward care. | 0.79 | 0.85 | 3.25 | 0.57 | 0.88 | 0.87 |
| 24 | The medical staff adjusted the plan well throughout the process of care. | 0.63 | 0.71 | 2.07 | **0.45^d^** | 0.93 | 0.89 |
| 25 | The medical staff paid attention to various symptoms I felt and adjusted them well. | 0.70 | 0.79 | 2.50 | 0.59 | 0.79 | 0.76 |
| 26 | I was educated on using analgesic. | 0.62 | 0.67 | 1.68 | 0.64 | **1.33^e^** | **1.44^f^** |
| 27 | My family and I received psychological support from the medical staff. | 0.73 | 0.79 | 2.52 | 0.67 | 1.02 | 0.98 |
| 28 | The medical staff communicated smoothly with me and my family. | 0.74 | 0.80 | 2.57 | 0.61 | 0.94 | 0.92 |
| 29 | The medical staff provided support to me and my family to overcome social crisis. | 0.77 | 0.82 | 2.83 | 0.50 | 0.84 | 0.85 |
| 30 | The medical staff provide support to me and my family to solve the spiritual concerns. | 0.65 | 0.70 | 1.70 | 0.63 | 1.02 | 1.05 |
| 31 | I was informed about spiritual service. | 0.60 | 0.65 | 1.43 | 0.62 | 1.19 | 1.26 |
| 32 | My religious beliefs and those of my family were respected. | **0.51^a^** | **0.55^b^** | **1.14^c^** | 0.56 | **1.58^e^** | **1.66^f^** |
| 33 | The medical staff knew what I wanted. | 0.69 | 0.74 | 2.23 | **0.51^d^** | **0.98^e^** | **1.02^f^** |
| 34 | I was able to receive continuous care from the medical staff. | 0.77 | 0.86 | 3.34 | 0.42 | 0.64 | 0.60 |
| 35 | The services needed for my care are provided by experts in their field. | 0.70 | 0.78 | 2.42 | 0.59 | 1.01 | 0.97 |
| 36 | I was able to get care services in the places I wanted. | 0.77 | 0.83 | 2.80 | 0.54 | 0.86 | 0.85 |
| 37 | There was someone on the staff who helped me manage my schedule, including outpatient treatment. | 0.63 | 0.69 | 1.74 | **0.42^d^** | 1.17 | 1.15 |
| 38 | Medical care was immediately provided in a state of crisis. | 0.69 | 0.76 | 2.24 | 0.58 | 0.99 | 0.95 |
| 39 | The medical treatments I received were provided as well as I wanted. | 0.76 | 0.84 | 3.05 | **0.41^d^** | **0.63^e^** | **0.60^f^** |
| 40 | I was able to receive professional advice at any time via telephone counseling. | 0.64 | 0.68 | 1.71 | 0.61 | **1.37^e^** | **1.37^f^** |
| 41 | Outpatient care and telephone counseling were done at the appointed time without delay. | 0.64 | 0.69 | 1.78 | 0.63 | 1.30 | 1.27 |
| 42 | The medical staff confirmed my regards even after the outpatient treatment. | 0.67 | 0.71 | 1.72 | 0.72 | **1.40^e^** | **1.44^f^** |
| 43 | I was able to have a conversation with medical staffs in a relaxed atmosphere. | 0.75 | 0.81 | 2.70 | 0.63 | 0.96 | 0.90 |
| 44 | I was able to receive outpatient care and telephone counseling with plenty of time. | 0.73 | 0.77 | 2.24 | 0.62 | 1.18 | 1.18 |

a.b. In case of DISCRIMINATION (Pearson / Polyseria), the value of 0.6 or less is shown in bold type and these items are removed.

c. A slope value of 1.6 or less is shown in bold type (the higher the score, the higher the degree of discrimination) and these items were excluded.

d. Factor loadings are shown in bold type with less than 0.5 in factor analysis results, and these items were removed.

e.f. In the case of INFIT / OUTFIT, a fit between 0.7 and 1.3 was interpreted as a good response, and other values were removed.

Appendix B. Quality Care Questionnaire –Palliative Care (QCQ-PC)

| No | Questions | Strongly  Agree | Agree | Disagree | Strongly  Disagree |
| --- | --- | --- | --- | --- | --- |
| Appropriate communication with health care professionals | | | | | |
| 1 | I am satisfied with the careful manner of medical staff |  |  |  |  |
| 2 | I am satisfied with the way of communication of medical staff |  |  |  |  |
| 3 | I was able to receive adequate care from medical staff |  |  |  |  |
| 4 | I have heard and understood an accurate description of the progress of my disease |  |  |  |  |
| 5 | The medical staff explained terms that I was curious about |  |  |  |  |
| 6 | I was able to receive the healthcare service I demanded |  |  |  |  |
| 7 | The medical staff support my decision on care plan |  |  |  |  |
| 8 | I have heard and understood an accurate description of my care plan |  |  |  |  |
| 9 | I was able to have a conversation with medical staff in a relaxed atmosphere |  |  |  |  |
| 10 | The medical staff paid attention to various symptoms I felt and adjusted them well |  |  |  |  |
| Discussing value of life and goals of care | | | | | |
| 11 | I was able to discourse with medical staff about the value of my life |  |  |  |  |
| 12 | I was able to recall what is important to achieve the values and goals of my life while discoursing with medical staff |  |  |  |  |
| 13 | I was able to express what my family and I expected from care |  |  |  |  |
| 14 | My care plans included the things I was able to try myself |  |  |  |  |
| 15 | I was able to receive adequate help from medical staff, while I was having difficulties in setting up specific goals related to care |  |  |  |  |
| 16 | My family and I received an education that is helpful to care |  |  |  |  |
| 17 | The medical staff suggested an adequate care plan in consideration of values of my life |  |  |  |  |
| 18 | I was able to modify my plan when my demand for treatment changed |  |  |  |  |
| 19 | The medical staff managed intermediate checkups to verify whether I could execute my goals |  |  |  |  |
| Support and counseling for needs of holistic care | | | | | |
| 20 | I was able to receive outpatient care and telephone counseling with plenty of time |  |  |  |  |
| 21 | The medical staff provide support to me and my family to solve spiritual concerns |  |  |  |  |
| 22 | The medical staff provided support to me and my family to overcome social crisis |  |  |  |  |
| 23 | The medical staff knew what I wanted |  |  |  |  |
| 24 | The medical staff communicated smoothly with me and my family |  |  |  |  |
| 25 | Outpatient care and telephone counseling were done at the appointed time without delay |  |  |  |  |
| 26 | My family and I received psychological support from medical staff |  |  |  |  |
| Accessibility and sustainability of care | | | | | |
| 27 | Services needed for my care are provided by experts in their respective fields |  |  |  |  |
| 28 | I was able to get care services at the locations I wanted |  |  |  |  |
| 29 | Medical care is immediately provided in a state of crisis |  |  |  |  |
| 30 | The medical staff periodically confirmed my goals and plans toward care |  |  |  |  |
| 31 | The decision on a healthcare plan was reflected by my family and my opinions |  |  |  |  |
| 32 | I understand the goal of care |  |  |  |  |
